# Ecological Preferences of Calliphoridae and Sarcophagidae (Diptera) in the Region Calabria (Southern Italy)

**DOI:** 10.3390/insects16090886

**Published:** 2025-08-25

**Authors:** Domenico Bonelli, Federica Mendicino, Francesco Carlomagno, Giuseppe Luzzi, Antonino Siclari, Federica Fumo, Erica Di Biase, Michele Mistri, Daniel Whitmore, Cristina Munari, Marco Pezzi, Teresa Bonacci

**Affiliations:** 1 Department of Biology, Ecology and Earth Sciences, University of Calabria, Via Pietro Bucci, 87036 Rende, Italy; domenico.bonelli@unical.it (D.B.); federica.mendicino@unical.it (F.M.); francesco.carlomagno@unical.it (F.C.); fumofede@gmail.com (F.F.); ericadibia@gmail.com (E.D.B.); teresa.bonacci@unical.it (T.B.); 2 Ente Parco Nazionale Lucano, Val d’Agri-Lagonegrese, Via Manzoni, 1, 85052 Marsico Nuovo, Italy; direttore@parcoappenninolucano.it; 3 Città Metropolitana di Reggio Calabria, Piazza Italia, 89100 Reggio Calabria, Italy; antonino.siclari@cittametropolitana.rc.it; 4 Department of Chemical, Pharmaceutical and Agricultural Sciences, University of Ferrara, Via Luigi Borsari 46, 44121 Ferrara, Italy; michele.mistri@unife.it (M.M.); cristina.munari@unife.it (C.M.); 5 Staatliches Museum für Naturkunde Stuttgart, Rosenstein 1, 70191 Stuttgart, Germany; daniel.whitmore@smns-bw.de; 6 Sistema Museale Universitario—SiMU, Sezione di Zoologia, University of Calabria, 87036 Rende, Italy

**Keywords:** Brachycera, community, Diptera, Italy, natural environment

## Abstract

**Simple Summary:**

Several trophic preferences have been described among Diptera belonging to the families Calliphoridae and Sarcophagidae, including necrophagous, saprophagous, and sometimes parasitic habits. Some species are of forensic and medical-veterinary importance, and among them, some exhibit synanthropic behavior, frequenting urban areas, livestock, and waste. A study on distribution, abundance and habitat preferences of species of Calliphoridae and Sarcophagidae was conducted in areas located in the Region Calabria (Southern Italy), including Aspromonte National Park, Sila National Park, Natural Regional Park of Serre, and a suburban area at the University of Calabria (Rende, Cosenza, Italy). A total of 14 species of Calliphoridae and 35 species of Sarcophagidae were collected, providing new data on local ecological and phenological preferences of these two families.

**Abstract:**

Diptera belonging to the families Calliphoridae and Sarcophagidae are known for their diversified trophic preferences and for their forensic and medical-veterinary relevance. The ecological preferences (distribution, abundance and habitat) of these two families were investigated along four years in the Region Calabria (Southern Italy) in 17 sampling sites located in four areas: the Aspromonte National Park, the Sila National Park, the Natural Regional Park of Serre, and a suburban area at the University of Calabria (Rende, Cosenza, Italy). A total of 39,537 individuals were collected, with 36,253 belonging to 14 species of Calliphoridae and 3284 belonging to 35 species of Sarcophagidae. The most abundant species among Calliphoridae was *Calliphora vomitoria* (Linnaeus, 1758); among Sarcophagidae, it was *Sarcophaga* (*Sarcophaga*) *croatica* Baranov, 1941. The highest species richness and abundance of Calliphoridae were observed in forest areas and those of Sarcophagidae in open and humid environments. The results also show a close association between the distribution of both families and environmental factors such as altitude, vegetation type, season, and temperature.

## 1. Introduction

The order Diptera includes approximately 160,000 species across 209 families [1], many of which possess medical, veterinary, forensic and ecological relevance [2,3]. They play a key role in trophic chains, pollination and economic, agronomic, silvicultural, and animal farming applications [4]. Many species are potential vectors of pathogens and can cause myiasis [5,6,7]. Diptera may exhibit high sinantropy, or asynanthropic behaviour, indicating a preference for natural habitats [8,9,10,11]. The families Calliphoridae (“blowflies”) and Sarcophagidae (“flesh flies”) include species that visit and colonize carcasses and human corpses, making them relevant in forensic entomology investigations [3]. The study of spatial and temporal distribution of Calliphoridae and Sarcophagidae, together with their ecological references, could provide valuable environmental insights [12] and useful information in forensic and medical-veterinary fields [13,14,15]. A considerable number of studies suggest that our understanding of their biotic and abiotic requirements should be extended [14,16]. It is known that several species of Calliphoridae are found in urban and suburban areas with broad environmental preference, while others are found in natural areas such as forests [11,13,14,15,17]. The family Sarcophagidae is usually associated with both natural and anthropogenic habitats [13,14,15,18,19,20,21,22,23]. Ecological data about these two families have already been reported in Europe [11,13,14,15,24,25,26,27,28,29,30,31,32,33,34,35,36]. Some studies have been conducted in Italy [11,35,36,37,38,39] and a few in Calabria [11,37,38,39]. Recent studies also focused on forensic and synanthropic Diptera [11,40,41,42,43,44]. The aim of this study is to provide information on the composition of dipteran communities in southern Italy, focusing on the families Calliphoridae and Sarcophagidae. Ecological preferences and the association of species of these two families with biotic and abiotic environmental factors were investigated.

## 2. Materials and Methods

### 2.1. Sampling Areas

**Figure 1:**
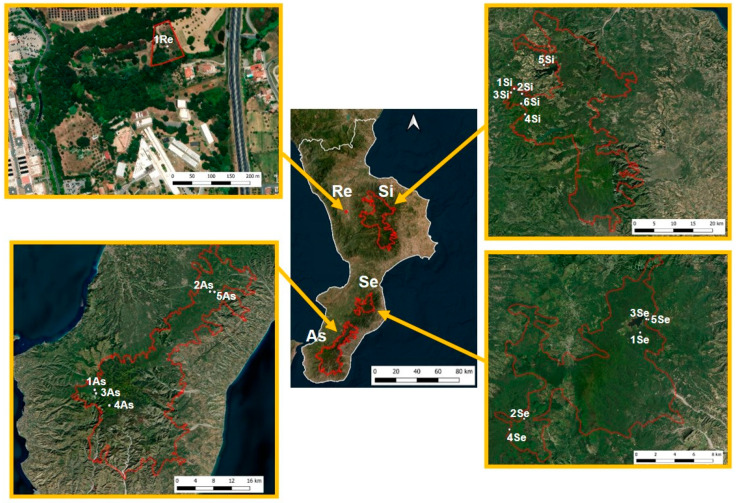
Areas and sites in the Region Calabria sampled for Diptera Brachycera. Abbreviations: Re, University of Calabria, Rende, 1 site; Si, Parco Nazionale della Sila (Sila National Park), 6 sites; As, Parco Nazionale dell’Aspromonte (Aspromonte National Park), 5 sites; Se, Parco Naturale Regionale delle Serre (Natural Regional Park of Serre), 5 sites. (Powered by Qgis software Prizren 3.3.4.2 version 2024).

Diptera belonging to the families Calliphoridae and Sarcophagidae were sampled within a monitoring project of Diptera Brachycera in three natural areas of the Calabrian Apennines: Aspromonte National Park in 2018–2019, Sila National Park in 2020–2021, and the Natural Regional Park of Serre in 2020–2021. A suburban area on the campus of the University of Calabria, Rende (Cosenza, Italy) was selected in 2020–2021 as the suburban site. For each natural area, three different habitats were selected: beech forests, pine forests and wetlands. The suburban site was characterized by a suburban meadow, while the selected habitats had different vegetation covers and were distributed across an altitudinal range from 220 to 1820 m a.s.l. A total of 17 sites were sampled (Figure 1), and their individual code, location, coordinates, altitude, environment and vegetation were previously reported [45].

### 2.2. Sampling Methods

During the monitoring project, 85 bait-bottle traps, prepared according to previous protocols [11,13,45], were set in the 17 chosen sites [45]. The bait-bottle traps were made from two 2 L clear plastic bottles, forming two chambers: the upper trapping chamber and the lower bait chamber. The bait chamber had holes for the flies to enter, attracted by the bait. The flies then remained trapped in the upper chamber. The bait chamber had two plastic containers, a 50 mL one with 25 g fresh bovine liver and 20 mL saturated NaCl solution, and a 100 mL one, with 50 g fresh bovine liver and 40 mL liquid protein bait (Dacus trap^®^, BioIberica, Barcelona, Spain) [11,13,45]. As with all food bait traps, its effectiveness may be affected by the dispersion of the bait odor, in turn influenced by environmental factors related to the location where the trap is positioned. Climatic data of the sampling periods were obtained by consulting the website of the Centro Funzionale Multirischi—ARPACAL (“Agenzia Regionale per la Protezione dell’Ambiente della Calabria”) (Catanzaro, Italy). Collected specimens were identified to the species level using taxonomic keys [17,18,19,46,47,48,49,50,51,52,53] and by comparison with specimens preserved at the Department of Biology, Ecology and Earth Sciences (DiBEST), University of Calabria. The specimens were stored in 80% ethanol and placed in the DiBEST collection.

### 2.3. Data Analysis

Calliphoridae and Sarcophagidae were analyzed for their phenology, abundance, distribution, ecological and habitat preferences using Past v.4.11 software [54] with statistical significance *p* < 0.05, and for preferences of species in relation to habitats by Indicator Value (IndVal) using RStudio v.2022.12.0 software [55]. Non-parametric multivariate analysis (PerMANOVA) [56] was carried out to study the distribution of the flies within the sampling areas. The Bray–Curtis similarity index was used to quantify the dissimilarity between Calliphoridae and Sarcophagidae. The random permutation method based on the Bray–Curtis similarity matrix was applied. To determine whether there were significant differences in the composition of communities, a PerMANOVA analysis was conducted with statistical significance *p* < 0.05, and post hoc Bonferroni pairwise comparisons were performed. Multiple comparisons (to adjust *p*-values and control the Type I error rate) were performed using the Bonferroni correction. Canonical Correspondence Analysis (CCA) was carried out to verify the main interrelationships between the species and the following environmental variables: altitude, grazing degree, human activities degree, shade degree, forest cover index, average seasonal temperature, and average seasonal rainfall (Table 1) [13,57,58]. For this analysis, we considered the following classification: suburban area (characterized by grass cover), forest habitat (including pine and beech forests), and wetland area. The species were codified as CAL_n for Calliphoridae and SAR_n for Sarcophagidae (Table A1 and Table A2). For Calliphoridae, species with an abundance lower than 0.1% were excluded from the analysis, and for Sarcophagidae, species with an abundance lower than 1% were not considered. Environmental variables and species from the two families were plotted separately on the two axes for clarity.

The Indicator Value (IndVal) [59], which ranges from 0 (no indication) to 1 (perfect indication), was used to assess the frequency of species in the areas and their preferences for vegetation and/or habitat type. The analysis was performed to describe possible exclusivity or fidelity of species to different groups: vegetation (beech forest, pine forest, wetland and suburban meadow) and habitat type (open areas: wetland and suburban meadow; closed areas: beech forest and pine forest) [60]. Species with an abundance of less than 0.1% were excluded from the analysis.

## 3. Results

### 3.1. Environmental Data

Meteorological data were collected from eight weather stations (ARPACAL: https://www.cfd.calabria.it/index.php/dati-stazioni/dati-storici, accessed on 10 January 2023) located near the 17 study sites (Figure 1; Table 2). During the study period, the highest mean temperature of 16 °C was recorded at the lowest elevation site (220 m a.s.l.), while the lowest mean temperature of 6.5 °C was observed at the higher elevation sites (1720 and 1820 m a.s.l.). The lowest recorded temperature was −14.9 °C (5Si; 6Si) in winter, and the highest recorded temperature was 42.7 °C (1Re) in summer. Precipitation, occurring as both rain and snow, was concentrated in the autumn and winter months. The highest average precipitation of 159.2 mm was recorded at 1As (912 m a.s.l.) and 2As (979 m a.s.l.), while the lowest average precipitation of 76.4 mm was recorded at 1Si (1140 m a.s.l.).

### 3.2. Abundances and Diversity

A total of 39,537 specimens belonging to the families Calliphoridae and Sarcophagidae were collected. Of these, 36,253 individuals (92%) were Calliphoridae, while 3284 individuals (8%) were Sarcophagidae. A total of 14 species were identified within Calliphoridae, and 35 species within Sarcophagidae. The most abundant species within the family Calliphoridae was *Calliphora vomitoria* (Linnaeus, 1758) with 16,422 specimens collected (45.50%), followed by *Calliphora vicina* Robineau-Desvoidy, 1830 with 10,856 specimens (29.94%), *Lucilia caesar* (Linnaeus, 1758) with 5151 (14.21%), and *Lucilia ampullacea* Villeneuve, 1922 with 2353 (6.49%). The most abundant species within the family Sarcophagidae was *Sarcophaga* (*Sarcophaga*) *croatica* Baranov, 1941 with 1495 specimens collected (45.52%), followed by *Sarcophaga* (*Sarcophaga*) *subvicina* Rohdendorf, 1937 with 481 (14.65%), *Sarcophaga* (*Liosarcophaga*) *portschinskyi* (Rohdendorf, 1937) with 194 (5.91%), and *Sarcophaga* (*Parasarcophaga*) *albiceps* Meigen, 1826 with 179 (5.45%) (Table A1 and Table A2). Concerning the species richness and abundance of the two families, the highest species richness and abundance of Calliphoridae were observed in forest areas, with a total of 14 species and 24,614 individuals recorded, while those of Sarcophagidae were observed in open and humid environments, with a total of 27 species and 1147 individuals recorded.

### 3.3. Seasonal Distribution

The PerMANOVA analysis showed significant differences in relative abundance among the seasons (Calliphoridae: F = 7.042, *p* = 0.0001, d.f. = 3; Sarcophagidae: F = 25.13, *p* = 0.0001, d.f. = 3). The corresponding post hoc Bonferroni pairwise test confirmed significant differences among the four seasons, except for a non-significant difference between spring and autumn in the seasonal distribution of Sarcophagidae (Table 3). A total of 18,275 specimens belonging to the Calliphoridae were sampled during the summer season (end of June and end of September), while 13,394 were sampled during the spring season (end of March and end of June). The lowest number of specimens was sampled during the winter season (end of December and end of March), with 4053 sampled in autumn and only 531 sampled in winter. The summer season was also characterized by the greatest species richness, with the recorded presence of all 14 sampled Calliphoridae species. In Sarcophagidae, the greatest abundance was recorded in summer (2356 specimens) and spring (554 specimens), followed by 369 in autumn and only 5 in winter, with the highest abundance (33 species) in summer. The Calliphoridae species *C. vicina* and *C. vomitoria* were the most abundant in all seasons. However, other species, such as *L. caesar* (4878 specimens), *L. ampullacea* (1630) and *Chrysomya albiceps* (Wiedemann, 1819) (190) showed high abundance in summer, while species such as *Lucilia sericata* (Meigen, 1826) showed high abundance (447) in spring, and *Calliphora rohdendorfi* (Grunin, 1966) increased in abundance (105) during the autumn season.

Sarcophagidae species were predominantly sampled during the summer season, with *S. croatica* (1116 specimens), *S. subvicina* (378), *S. albiceps* (132) and *S. portschinskyi* (131) being the most abundant in summer. *Sarcophaga croatica* was the species found in all sampled seasons.

### 3.4. Altitudinal Gradient

An analysis of the distribution of the Calliphoridae and Sarcophagidae species along altitudinal gradients was conducted. According to ecological standards [61], an altitudinal value was assigned to each area. The first altitudinal range, named “Hilly”, was set between 0 and 1000 m a.s.l.; the second, “Submontane” was set between 1000 and 1300 m a.s.l.; and the third, “Montane” was set between 1300 and 2000 m a.s.l. The Calliphoridae were found to be prevalent at almost all sites, with the highest recorded abundance occurring at altitudes of 912 m in 5As (3514 specimens) and 1060 m in 1Se (3564 specimens). In relation to altitudinal ranges, Calliphoridae showed no significant difference in abundance (F = 1.265, *p* = 0.2532, d.f. = 2) but significant differences in species distribution along the altitudinal gradient (F = 3.385, *p* = 0.0004, d.f. = 2). The corresponding post hoc Bonferroni pairwise test confirmed significant differences between Hilly and Submontane (*p* = 0.0081), and between Hilly and Montane (*p* = 0.0009), but showed no significant differences between Submontane and Montane (*p* = 0.1149) (Table 4). The species *C. vicina* and *C. vomitoria* were the most prevalent in all altitudes. Other species, including *L. sericata* and *Ch. albiceps*, were recorded at altitudes below 1000 m, while *C. rohdendorfi* was sampled from elevations above 1000 m. (Table A1). The family Sarcophagidae exhibited highly significant differences in relation to the altitude gradient (F = 7131, *p* = 0.0002, d.f. = 2). Abundance of Sarcophagidae species showed a significant difference between altitudinal gradient (F = 6.213, *p* = 0.0001, d.f. = 2). Post hoc Bonferroni pairwise tests confirmed the significant differences between Hilly and Montane (*p* = 0.003), as well as between Submontane and Montane (*p* = 0.0084), but no significant difference between Hilly and Submontane (*p* = 0.1038) (Table 4). Additionally, the species richness of Sarcophagidae exhibited significant variations with increasing altitude. The two lowest altitude sites (1Re and 5As) showed the greatest species richness while at the highest altitude site (1820 m, 4Si), only *S. croatica* was sampled.

### 3.5. Habitat Preferences

Concerning the family Calliphoridae, no differences were detected in abundance and distribution between open and closed habitats (F = 0.57; *p* = 0.6397; d.f. = 1), whereas for the family Sarcophagidae, there were significant differences in abundance and distribution between open and closed habitats (F = 7.8; *p* = 0.0007 d.f.= 1; Bonferroni pairwise test, *p* = 0.0013). The abundance and distribution of Calliphoridae species collected showed significant differences among different habitats (F = 6.811, *p* = 0.0001, d.f. = 1; Bonferroni pairwise test, *p* = 0.0001).

For the species of the family Sarcophagidae, a significant difference was also found in abundance and distribution of species (F = 7.125, *p* = 0.001, d.f. = 1; Bonferroni pairwise test, *p* = 0.0011), with adapted species observed exclusively in open habitats (Table A2).

About the different vegetation types studied (beech forest, pine forest, wetland and suburban meadow), no significant differences were found in the abundance of the family Calliphoridae (F= 0.4692, *p* = 0.9096, d.f. = 3). However, significant differences for the species were found between vegetation types (F = 3.736, *p* = 0.0001, d.f. = 3). The Bonferroni pairwise test confirmed significant differences between forest and wetland habitats, as well as between forest and suburban meadow habitats, while no significant differences were found between wetlands and suburban meadow habitats (Table 5). The abundance of the family Sarcophagidae showed clear significant differences in preferences about vegetation types (F = 3.788, *p* = 0.0017, d.f. = 3). Similar significant differences were found among the species of this family (F = 3.734, *p* = 0.0006, d.f. =3) in the distribution between beech forests and wetlands, as well as between forest and suburban meadow habitats (Table 5).

### 3.6. IndVal

The potential preferences of the species were also analyzed in relation to the habitats. For the initial analysis, the individual types of vegetation that characterized the sites were considered and subsequently grouped into two categories, “open” (wetland and suburban meadow) and “closed” (beech forest and pine forest) habitats). No significant differences were detected among groups in relation to habitat types. For Calliphoridae, three species were found to be indicative: *L. sericata* (IndVal = 0.987, *p* = 0.0030) and *Protocalliphora azurea* (Fallén, 1817) (IndVal = 0.904, *p* = 0.0426) showed a preference for wetlands and suburban meadows, and were thus indicative species for open habitats. *Calliphora rohdendorfi* (IndVal = 0.957, *p* = 0.0014) was associated with pine forests and beech forests, and was thus indicative for closed habitats. Within Sarcophagidae, *Ravinia pernix* (Harris, 1780) (IndVal = 0.723, *p* = 0.0374) and *Sarcophaga* (*Bercaea*) *africa* (Wiedemann, 1824) (IndVal = 0.721, *p* = 0.0382) were found to be associated with open habitats.

### 3.7. CCA

**Figure 2:**
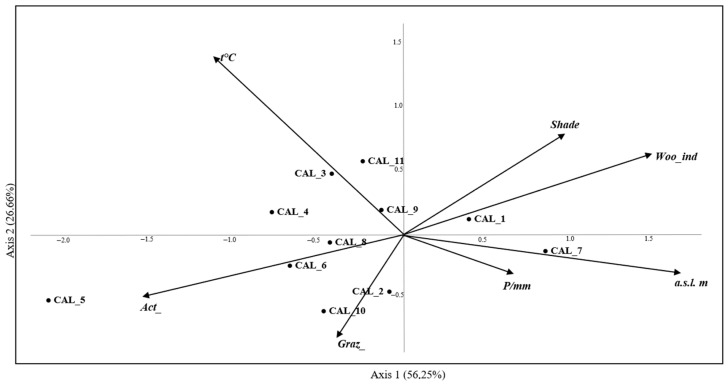
The canonical correspondence analysis (CCA) performed on a matrix of species from the family Calliphoridae collected from the sites listed in Pezzi et al. [45] and another matrix of the environmental variables listed in Table 1. Arrows represent the environmental variables. For abbreviations of variables, see Table 1. Species abbreviations: CAL_1, *Calliphora vomitoria*; CAL_2, *C. vicina*; CAL_3, *Lucilia caesar*; CAL_4, *L. ampullacea*; CAL_5, *L. sericata*; CAL_6 *Chrysomya albiceps*; CAL_7, *C. rohdendorfi*; CAL_8, *Protocalliphora azurea*; CAL_9, *Melinda gentilis* Robineau-Desvoidy, 1830; CAL_10, *Onesia floralis* Robineau-Desvoidy, 1830; CAL_11, *Eurychaeta muscaria* (Meigen, 1826).

**Figure 3:**
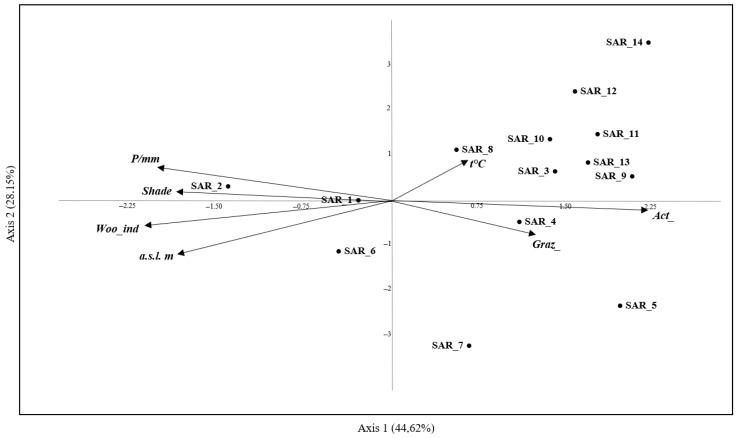
The canonical correspondence analysis (CCA) performed on a matrix of species from the family Sarcophagidae collected from the sites listed in Pezzi et al. [45] and another matrix of the environmental variables listed in Table 1. Arrows represent the environmental variables. For abbreviations of variables, see Table 1. Species abbreviations: SAR_1, *Sarcophaga croatica*; SAR_2, *S. subvicina*; SAR_3, *S. portschinskyi*; SAR_4, *S. albiceps*; SAR_5, *Ravinia pernix*; SAR_6, *S. caerulescens*; SAR_7, *Angiometopa falleni*; SAR_8, *S. haemorrhoa*; SAR_9, *S. incisilobata*; SAR_10, *S. teretirostris*; SAR_11, *S. africa*; SAR_12, *S. aratrix*; SAR_13, *S. argyrostoma*; SAR_14, *S. melanura*.

For CCA of the family Calliphoridae, the eigenvalues of the first two axes of CCA were 0.23 and 0.11, accounting for 56.25% and 26.66% of the total variance, respectively (Figure 2). The third axis explained 11.33% of the variance, so only the first two axes were used in subsequent plots. As shown in Figure 2, of the 11 species analyzed, CAL_7 (*C. rohdendorfi*) and CAL_1 (*C. vomitoria*) were primarily concentrated around the origin of the axes. *Calliphora rohdendorfi* showed a positive relationship with altitude, shade, and forest cover index. *Calliphora vomitoria* was influenced by the forest cover index, but less from altitude. The graph indicates that human activities have little impact on the distribution of most species. The variables shade, altitude, and forest cover index may negatively affect species distribution. *Lucilia sericata* (CAL_5) was the only species with a different distribution pattern. The rest of the Calliphoridae species were concentrated around the origin of the axes. For Sarcophagidae, the eigenvalues of the first two axes of CCA were 0.21 and 0.13, accounting for 44.62% and 28.15% of the total variance, respectively, with the third axis measuring 12.00% of the variance (Figure 3).

The analysis demonstrated heterogeneity in the ecological responses of the Sarcophagidae species to the environmental variables considered. Based on Figure 3, the species examined appeared gathered in two groups: the first group, located to the upper right of the graph, including *Sarcophaga* (*Heteronychia*) *haemorrhoa* Meigen, 1826 (SAR_8), *Sarcophaga* (*Liosarcophaga*) *teretirostris* Pandellé, 1896 (SAR_10), *S. portschinskyi* (SAR_3), *Sarcophaga* (*Liopygia*) *argyrostoma* (Robineau-Desvoidy, 1830) (SAR_13), *Sarcophaga* (*Thyrsocnema*) *incisilobata* Pandellé, 1896 (SAR_9), *S. africa* (SAR_11), *Sarcophaga* (*Rosellea*) *aratrix* Pandellé, 1896 (SAR_12) and *Sarcophaga* (*Helicophagella*) *melanura* Meigen, 1826 (SAR_14), showed an association with temperature and human activities. *Sarcophaga albiceps* (SAR_4) was found to be closely associated with open, grazed pastures. *Ravinia pernix* (SAR_5) and *Angiometopa falleni* Pape, 1986 (SAR_7) exhibited a similar preference for open, grazed pastures. On the other hand, the second group, positioned on the left of the graph, included *S. croatica* (SAR_1), *Sarcophaga (Robineauella) caerulescens* Zetterstedt, 1838 (SAR_6) and *S. subvicina* (SAR_2). The first two species did not show any significant associations with the investigated variables. The third species, *S. subvicina* (SAR_2), differed from the other two for the preference for humid and shady environments, such as forest habitats.

## 4. Discussion

Diptera represent a large taxonomic group that includes families considered harmful to animals, humans, and economic activities. They play a key role in the food chains of many ecosystems, both terrestrial and aquatic [62]. Investigations on Calliphoridae and Sarcophagidae have been limited regarding their temporal and spatial distribution [11,13,14,15,24,25,26,29,30,31,32,33,34,35,36], and many studies focused on species of forensic and medical-veterinary importance [5,44,63,64,65,66,67,68].

The analyses performed in this study on Calliphoridae in the Region Calabria (Southern Italy) showed no significant effects of altitude on the total abundance of the family (F = 1.265, *p* = 0.2532, d.f. = 2), but the PerMANOVA analysis indicated a preference for forest environments over open ones. This suggests a greater adaptation of these dipterans to more stable microclimatic conditions and to the availability of substrates for larval development. Furthermore, seasonality has been shown to significantly influence the activity of these insects, with peaks in abundance occurring during the spring–summer months, indicating a strong dependence on optimal thermal conditions. However, concerning the altitude, several species of Calliphoridae showed a wide altitudinal and environmental distribution. Some species appear to be associated with altitude, forest environments, and specific altitudinal and thermal ranges. According to previous studies in Calabria [11], *C. vicina* and *C. vomitoria* were found to occur in all seasons and sites, making them the most representative species of this family. Both are abundant through the year, as also documented in the literature [11,13,14,15,28,29,30,33,69]. Both species are thermophobic and show a preference for humid habitats that are minimally exposed to sunlight and have low temperatures [24], as evidenced by their abundance in shaded sites during the summer. *Calliphora vomitoria* shows a preference for forest environments in comparison to *C. vicina*, in accordance with the literature [13,24,29,70]. The altitude range was not a determining factor for the distribution of *C. vicina* and *C. vomitoria*, as previously found in Sicily [36]. These two species are apparently adapted to mountain conditions, with significantly higher abundance at higher altitudes compared to other species [26,33]. Regarding *C. rohdendorfi*, a new faunal record for Italy and Southern Europe [45], this species showed a significantly association with forest environments and high altitude, as shown by IndVal and CCA analyses. Current knowledge of the biology and ecology of this species is limited and requires further studies.

Given its ecological characteristics, *C. rohdendorfi* could be relevant for conservation of forest communities and ecosystems, and it may serve as an early indicator of structural changes induced by climate change, as detected for other species [71].

With regard to the genus *Lucilia*, the three species recorded (*L. ampullacea*, *L. caesar*, *L. sericata*) are of forensic importance [5,64,72,73]. They are more abundant in the summer season, although there are some differences in abundance with respect to altitude. *Lucilia caesar* was the dominant species in the genus *Lucilia*. In previous studies, it has been reported as a thermophilic species, distributed at low and medium altitudes [11,13,17,24,26,33,70]. Our data showed that *L. caesar* prefers forests located across a broad altitudinal range (until 1820 m in this study, but with few samples), and, as a cosmopolitan species, is strongly influenced by the thermal range and by the season, preferring medium–high temperatures. *Lucilia caesar* was found predominantly in shaded environments rather than open ones, in accordance with previous studies [11,13,14,15,26,29,30]. *Lucilia ampullacea*, although less abundant than *L. caesar*, was found at all sampled sites, with the highest densities recorded in suburban environments, in agreement with previous studies [11,13,15]. In our observations, *L. ampullacea* exhibits a temporal–spatial distribution similar to that of *L. caesar*, showing increased abundance at low altitudes during the spring and autumn seasons. Conversely, during the summer, the species was more prevalent at higher altitudes and in forest environments, confirming its thermophilic behavior [11,15,69]. *Lucilia sericata*, a synanthropic species, is also associated with grazing environments hosting animals, such as sheep [13,72,74], and in Calabria, it was detected in association with urban and suburban environments [11]. In our study, *L. sericata* exhibited a higher prevalence at low altitude in suburban areas (220 m a.s.l). This species can be detected in higher areas characterized by human activity or grazing (1140 m a.s.l.). In a previous study in Sicily, *L. sericata* showed an apparent preference for low-altitude sites [36].

The IndVal analysis confirmed the association of *L. sericata* with open environments, identifying it as an indicator species for such habitats. The CCA further highlighted the synanthropic nature of the species, emphasizing its close link with human activities. These results are in agreement with those of previous studies [11,13,14,24,26,30,75,76]. The phenology of *L. sericata* exhibited a peak in abundance during spring and autumn, with a decline during summer and absent in winter. This distribution pattern does not agree with the results of previous studies, which generally associate this species with warmer periods (summer), but agrees with those showing its presence in open habitats [11,13,14,24,26,30,75,76]. This species has also been found in winter in indoor environments closely associated with human activities [33]. *Chrysomya albiceps* is a native tropical and subtropical African species [5,47] that shows a pattern of global expansion, with its current distribution almost cosmopolitan [77]. This species is reported as heliophilic and thermophilic [78]. In addition, *Ch. albiceps* is an important forensic indicator [79], and it can also cause severe primary and secondary myiasis [5]. The results of this study show a clear preference of *Ch. albiceps* for open, low-altitude environments and warm temperatures, as previously documented [14,24,26,33,34,35,40]. The species was always sampled at altitudes below 1370 m, except for a single individual recorded at 1370 m. The highest activity of *Ch. albiceps* was recorded during the summer and autumn months, with a preference for open areas with grazing and human activities, as previously documented [40]. This seasonal distribution was not reported in previous observations in Calabria [11], where the species results were rare, with only few samples collected in similar habitats. The species exhibits a seasonal trend similar to that previously reported for the genera *Calliphora* and *Lucilia*, with high abundance at low altitudes in spring and a shift to higher altitudes in summer.

*Protocalliphora azurea*, a parasite of bird nests [17], demonstrated a preference for open environments as shown by the IndVal analysis, with a peak in abundance during the summer months. The remaining Calliphoridae species were found in lower abundances. *Melinda gentilis* and *Melinda viridicyanea* (Robineau-Desvoidy, 1830), both snail parasites and potential forensic indicators [17,28,29], were found across a broader range of habitats, albeit at lower abundance, with a preference for the spring and summer months. Specifically, *M. gentilis* exhibited higher abundance compared to *M. viridicyanea*. *Eurychaeta muscaria* and *O. floralis*, both flower visitors and snail parasites [17], displayed similar distribution and abundance across a wide altitudinal range, with no clear preferences. Finally, *Bellardia viarum* (Robineau-Desvoidy, 1830), an earthworm parasite [17], and *Protophormia terraenovae* (Robineau-Desvoidy, 1830), a species of forensic interest [80,81], were found exclusively during the summer months.

The analyses of Sarcophagidae showed a clear preference for open, sun-exposed, and human-inhabited environments, supporting the findings of previous studies in Europe [13,14,15,18,19,20,21,22,23] and South America [12,16,82,83,84]. Statistical analyses (PerMANOVA and IndVal) confirmed this association, suggesting that these dipterans are strongly adapted to specific microclimatic conditions. Based on abundance of species of the family Sarcophagidae, the species can be divided into three groups. The first group includes *S. croatica* and *S. subvicina*, the dominant species, as represented on the left of the CCA chart. These species results are ubiquitous in this study, showing a wide and uniform distribution along the considered altitudinal gradient. The activity of these Sarcophagidae was recorded from late spring to late autumn, with a peak in summer, even at the highest altitudes. Although *S. croatica* showed lower abundance in beech forests, likely due to the altitude of the sites, its distribution was broad, as observed in the CCA and confirmed in previous studies [22,23].

The identification of *S. croatica* is often difficult because of its close morphological resemblance to *Sarcophaga* (*Sarcophaga*) *variegata* (Scopoli, 1763), a similar species [22,39]. *Sarcophaga croatica* is widespread in Italy, where it replaces *S. variegata* in the central–southern regions and in Sicily [39]. *Sarcophaga subvicina*, which is also more abundant in summer, showed a clear preference for shaded forest environments in this study, as observed in the CCA. However, the species was also found in urban, suburban, and sunny open areas [13,15], and was reported as a parasitoid of earthworms [19]. The second group consists of species predominantly located on the right side of the CCA chart, indicating a high preference for open habitats and summer temperatures, with a concentrated distribution at low altitude, as confirmed by similar studies [13,21]. *Angiometopa falleni*, *S. aratrix* and *S. haemorrhoa* were primarily identified at medium–low altitudes during the summer months in open and grazing areas. *Sarcophaga incisilobata* and *S. teretirostris* also showed a marked affinity for such environments, with *S. incisilobata* reported also in urban areas [15]. *Sarcophaga africa*, *S. argyrostoma* and *S. melanura* exhibited a marked synanthropic behavior, with a tendency to inhabit open, suburban, and natural areas in close proximity to anthropic activity, as well as humid and grazing areas. Previous studies showed that these species have a peak of abundance during the warmer periods [13,15,20,21]. IndVal and CCA highlighted a close association between *S. africa* and open environments, confirming previous findings [13,15,21]. Furthermore, according to the data of this study, *S. aratrix* and *S. caerulescens* appeared more generalist compared to other species, as found in other studies [13,15,21]. In one of these studies, *S. caerulescens* was found to be present at high altitudes during the summer months [21]. In our study, *S. portschinskyi* and *S. albiceps* were also found in open areas at low altitudes, with *S. portschinskyi* showing a different presence in altitude according to the season. In our study, this condition was also observed for some species of Calliphoridae, supporting previous data [26]. *Sarcophaga albiceps* has been documented to prefer open and sunny environments [21,22], often in anthropized or grazing areas, as shown by the CCA in our study, but it has also been observed in urban areas [15]. Finally, IndVal analysis identified *R. pernix* as indicative of open environments, supporting a previous study on the species [21]. Some species of Sarcophagidae, including *S. caerulescens*, *S. africa*, *S. argyrostoma*, *S. melanura*, *S. portschinskyi*, *S. albiceps* and *R. pernix*, have been reported to be of forensic and medico-veterinary interest [20,21,43,66]. The third group includes species with lower abundance. Some of them species, *Sarcophaga* (*Heteronychia*) *vagans* Meigen, 1826, *Sarcophila latifrons* (Fallén, 1817), *Sarcophaga* (*Heteronychia*) *filia* Rondani, 1860, *Sarcophaga* (*Helicophagella*) *hirticrus* Pandellé, 1896, *Sarcophaga* (*Myorhina*) *nigriventris* Meigen, 1826, *Sarcophaga* (*Liopygia*) *crassipalpis* Macquart, 1839, *Sarcophaga* (*Heteronychia*) *depressifrons* Zetterstedt, 1845 and *Agria affinis* (Fallén, 1817) were primarily found in open environments at low altitude, often associated with human activity and grazing, confirming previously described distributions [13,15,21]. Other species of this group, such as *Sarcophaga* (*Liosarcophaga*) *aegyptica* Salem, 1935, *Sarcophaga* (*Liosarcophaga*) *jacobsoni* (Rohdendorf, 1937), *Sarcophaga* (*Heteronychia*) *consanguinea* Rondani, 1860, *Sarcophaga* (*Liosarcophaga*) *tibialis* Macquart, 1851, and *Sarcophaga* (*Heteronychia*) *siciliana* (Enderlein, 1928), were caught almost exclusively in the low-altitude site located in a suburban area. Only one specimen was collected of the following species: *Sarcophaga* (*Pandelleisca*) *similis* Meade, 1876, *Sarcophaga* (*Heteronychia*) *dissimilis* Meigen, 1826, and *Macronychia polyodon* (Meigen, 1824).

It is important to note that *S. aegyptica, S. crassipalpis*, *S. similis*, and *S. tibialis* are of forensic and medical-veterinary interest [20,42,66,85].

## 5. Conclusions

This study provides new insights into the ecological preferences and distribution of species from the families Calliphoridae and Sarcophagidae. Our findings demonstrate a close association between the distribution of these families and environmental factors, including altitude, vegetation type, and temperature. The analyses demonstrated a general difference in the altitudinal distribution of the families Calliphoridae and Sarcophagidae. While the abundance of Calliphoridae remained relatively constant along the altitude gradient, the abundance of Sarcophagidae exhibited a marked decrease with increasing altitude. The data can be attributed to the differing responses of the two families to thermal variations. Some species of Calliphoridae are known to be able to colonize a wide altitudinal range along the seasons. Sarcophagidae, however, being highly thermophilic and heliophilic, exhibited a more limited distribution, with a higher concentration observed at lower altitudes. These results could be useful for further investigations into the ecological dynamics of these insects, offering valuable perspectives on how environmental changes may affect these species in Mediterranean ecosystems. Moreover, some of these species could be relevant as bioindicators for climate change, in relation to shifts in altitudinal and habitat preferences.

## Figures and Tables

**Table 1:** Environmental variables used in canonical correspondence analysis (CCA): altitude (m a.s.l.), grazing degree, human activities degree, shade degree, forest cover index (the proportion of the area under forest relative to the total area of a given territory, expressed as a percentage), average seasonal temperature, (e.g., average, minimum, and maximum seasonal temperatures), and average seasonal rainfall (in millimeters).

Variables	Code	Description
Altitude (220 m to 1820 m)	*a.s.l. m*	Altitudinal gradient of the 17 sampling sites.
Grazing degree (0 to 2)	*Graz_*	Grazing activity was estimated through direct observation and the manipulation of traps at and around the sites. The following scale was used to categorize the level of grazing activity observed: 0 = no activity; 1 = sporadic presence of grazing animals during a few short periods throughout the year; 2 = grazing activity occurring for approximately half of the year.
Human activities degree (1 to 4)	*Act_*	The presence of human activity, population centers, and primary roads with moderate or intense traffic was observed around sites. This was assessed using satellite imagery with 500 × 500 m grids, as outlined in Hwang and Turner [13].
Shade degree (0 to 4)	*Shade*	The approximate level of shade around the traps was visually assessed based on tree cover, using the following scale: 0 = fully exposed; 1 = partially shaded; 2 = moderately shaded; 3 = heavily shaded; 4 = fully shaded and constant.
Forest cover index (0 to 100%)	*Woo_ind*	The proportion of the area covered by forest relative to the total area of a given territory, expressed as a percentage. The calculations were performed using the wooded surface layers of the areas under study (Qgis software).
Average seasonal temperature (°C)	*t* °C	The seasonal average temperature in Celsius degrees for each site was obtained through consultations with ARPCAL climate monitoring stations.
Average seasonal rainfall (P/mm)	P/*mm*	The average seasonal rainfall for each site was obtained through consultations with ARPCAL climate monitoring station.

**Table 2:** Temperatures (T) (minimum, maximum and mean) and mean precipitation (P) recorded at eight weather stations (ARPACAL: https://www.cfd.calabria.it/index.php/dati-stazioni/dati-storici, accessed on 10 January 2023) located near the 17 study sites. Abbreviation of sites as in Pezzi et al. [45].

Sites	Minimum T (°C)	Maximum T (°C)	Mean T (°C)	Mean P (mm)
2As, 5As	−6.8	32.7	11.6	159.2
1As, 3As, 4As	−6.6	32.2	11	137.5
5Si	−12.6	35.9	9.3	76.4
1Si, 2Si, 3Si	−14.3	31.5	7.17	100.6
4Si, 6Si	−14.9	28	6.5	101.2
1Se, 3Se, 5Se	−6.8	36.4	12	108.3
2Se, 4Se	−5.4	37.1	12	116.9
1Re	−2.1	42.7	16	79.1

**Table 3:** Bonferroni pairwise test for seasons.

	Calliphoridae	Sarcophagidae
	*p*-Value	*p*-Value
Spring vs. Summer	0.0144	0.0006
Spring vs. Autumn	0.0024	1
Spring vs. Winter	0.0006	0.0012
Summer vs. Autumn	0.0006	0.0006
Summer vs. Winter	0.0006	0.0006
Autumn vs. Winter	0.0384	0.0054

**Table 4:** Bonferroni pairwise test for the altitudinal gradient: Hilly (0–1000 m a.s.l.), Submontane (1000–1300 m a.s.l.), and Montane (1300–2000 m a.s.l.).

	Calliphoridae	Sarcophagidae
	*p*-Value	*p*-Value
Hilly vs. Submontane	0.0081	0.1038
Hilly vs. Montane	0.0009	0.0003
Submontane vs. Montane	0.1149	0.0084

**Table 5:** Bonferroni pairwise test for vegetation type: beech forest (BF), pine forest (PF), suburban meadow (SM), wetland (WL).

	Calliphoridae	Sarcophagidae
	*p*-Value	*p*-Value
WL vs. PF	0.0024	0.303
WL vs. BF	0.0264	0.0264
WL vs. SM	0.0312	0.3234
SM vs. PF	0.0012	0.0462
SM vs. BF	0.0018	0.003
BF vs. PF	1	1

## Data Availability

The original contributions presented in this study are included in the article. Further inquiries can be directed to the corresponding author [M.P.].

## Appendix A

**Table A1:** Checklist of Calliphoridae species with identification CCA code, abundance (number of individuals collected) (n), relative abundance (%), and abundance in different vegetation types: beech forest (BF), pine forest (PF), suburban meadow (SM), wetland (WL). Coverage: closed habitat (C); open habitat (O).

CCA Code	Species	n	(%)	Vegetation Type				Coverage	
				WL	PF	BF	SM	C	O
**CAL_1**	*Calliphora vomitoria* (Linnaeus, 1758)	16,422	45.50%	2539	6859	6895	129	13754	2668
**CAL_2**	*Calliphora vicina* Robineau-Desvoidy, 1830	10,856	29.94%	4059	2590	3040	1167	5630	5226
**CAL_3**	*Lucilia caesar* (Linnaeus, 1758)	5151	14.21%	776	2219	1324	832	3543	1608
**CAL_4**	*Lucilia ampullacea* Villeneuve, 1922	2353	6.49%	343	838	445	727	1283	1070
**CAL_5**	*Lucilia sericata* (Meigen, 1826)	731	2.02%	140	11	9	571	20	711
**CAL_6**	*Chrysomya albiceps* (Wiedemann, 1819)	317	0.87%	160	78	24	55	102	215
**CAL_7**	*Calliphora rohdendorfi* (Grunin, 1966)	134	0.37%	\	56	78	\	134	\
**CAL_8**	*Protocalliphora azurea* (Fallén, 1817)	93	0.26%	68	11	6	8	17	76
**CAL_9**	*Melinda gentilis* Robineau-Desvoidy, 1830	67	0.18%	13	24	26	4	50	17
**CAL_10**	*Onesia floralis* Robineau-Desvoidy, 1830	46	0.13%	23	15	3	5	18	28
**CAL_11**	*Eurychaeta muscaria* (Meigen, 1826)	46	0.13%	4	25	13	4	38	8
**\**	*Melinda viridicyanea* (Robineau-Desvoidy, 1830)	30	0.08%	9	10	10	1	20	10
**\**	*Bellardia viarum* (Robineau-Desvoidy, 1830)	5	0.01%	1	2	2	\	4	1
**\**	*Protophormia terraenovae* (Robineau-Desvoidy, 1830)	2	0.01%	1	\	1	\	1	1
	**Total**	**36,253**	**100%**	**8136**	**12,738**	**11,876**	**3503**	**24,614**	**11,639**
		**N° species→**		**13**	**13**	**14**	**11**	**14**	**13**

**Table A2:** Checklist of Sarcophagidae species with identification CCA code, abundance (number of individuals collected) (n), relative abundance (%) and abundance in different vegetation types: beech forest (BF), pine forest (PF), suburban meadow (SM), wetland (WL). Coverage: closed habitat (C); open habitat (O).

CCA Code	Species	n	(%)	Vegetation Type				Coverage	
				WL	PF	BF	SM	C	O
**SAR_1**	*Sarcophaga* (*Sarcophaga*) *croatica* Baranov, 1941	1495	45.52%	446	508	207	334	715	780
**SAR_2**	*Sarcophaga* (*Sarcophaga*) *subvicina* Rohdendorf, 1937	481	14.65%	66	224	123	68	347	134
**SAR_3**	*Sarcophaga* (*Liosarcophaga*) *portschinskyi* (Rohdendorf, 1937)	194	5.91%	81	23	2	88	25	169
**SAR_4**	*Sarcophaga* (*Parasarcophaga*) *albiceps* Meigen, 1826	179	5.45%	95	13	16	55	29	150
**SAR_5**	*Ravinia pernix* (Harris, 1780)	124	3.78%	119	2	\	3	2	122
**SAR_6**	*Sarcophaga* (*Robineauella*) *caerulescens* Zetterstedt, 1838	103	3.14%	44	30	23	6	53	50
**SAR_7**	*Angiometopa falleni* Pape, 1986	89	2.71%	70	19	\	\	19	70
**SAR_8**	*Sarcophaga* (*Heteronychia*) *haemorrhoa* Meigen, 1826	88	2.68%	38	10	\	40	10	78
**SAR_9**	*Sarcophaga* (*Thyrsocnema*) *incisilobata* Pandellé, 1896	77	2.34%	56	2	\	19	1	76
**SAR_10**	*Sarcophaga* (*Liosarcophaga*) *teretirostris* Pandellé, 1896	54	1.64%	17	7	2	28	9	45
**SAR_11**	*Sarcophaga* (*Bercaea*) *africa* (Wiedemann, 1824)	45	1.37%	14	\	\	31	\	45
**SAR_12**	*Sarcophaga* (*Rosellea*) *aratrix* Pandelle, 1896	44	1.34%	9	\	\	35	\	44
**SAR_13**	*Sarcophaga* (*Liopygia*) *argyrostoma* (Robineau-Desvoidy, 1830)	39	1.19%	16	\	\	23	\	39
**SAR_14**	*Sarcophaga* (*Helicophagella*) *melanura* Meigen, 1826	35	1.07%	1	\	\	34	\	35
**\**	*Sarcophaga* (*Liosarcophaga*) *aegyptica* Salem, 1935	26	0.79%	\	\	\	26	\	26
**\**	*Sarcophaga* (*Heteronychia*) *filia* Rondani, 1860	26	0.79%	20	6	\	\	6	20
**\**	*Sarcophila latifrons* (Fallén, 1817)	26	0.79%	11	2	\	13	2	24
**\**	*Sarcophaga* (*Heteronychia*) *vagans* Meigen, 1826	24	0.73%	10	13	1	\	14	10
**\**	*Sarcophaga* (*Myorhina*) *nigriventris* Meigen, 1826	23	0.70%	16	7	\	\	7	16
**\**	*Sarcophaga* (*Liosarcophaga*) *jacobsoni* (Rohdendorf, 1937)	20	0.61%	1	\	1	18	1	19
**\**	*Sarcophaga* (*Helicophagella*) *agnata* Rondani, 1860	19	0.58%	4	11	4	\	15	4
**\**	*Sarcophaga* (*Heteronychia*) *consanguinea* Rondani, 1860	16	0.49%	\	1	\	15	1	15
**\**	*Sarcophaga* (*Heteronychia*) *vicina* Macquart, 1835	12	0.37%	2	3	2	5	5	7
**\**	*Sarcophaga* (*Helicophagella*) *hirticrus* Pandellé, 1896	11	0.33%	2	1	\	8	1	10
**\**	*Sarcophaga* (*Helicophagella*) *novercoides* Böttcher, 1913	11	0.33%	1	9	\	1	9	2
**\**	*Sarcophaga* (*Heteronychia*) *depressifrons* Zetterstedt, 1845	4	0.12%	4	\	\	\	\	4
**\**	*Sarcophaga* (*Liopygia*) *crassipalpis* Macquart, 1839	4	0.12%	\	\	\	4	\	4
**\**	*Sarcophaga* (*Liosarcophaga*) *tibialis* Macquart, 1851	4	0.12%	1	\	\	3	\	4
**\**	*Sarcophaga* (*Heteronychia*) *siciliana* (Enderlein, 1928)	2	0.06%	\	\	\	2	\	2
**\**	*Sarcophaga* (*Myorhina*) *socrus* Rondani, 1860	2	0.06%	\	2	\	\	2	\
**\**	*Sarcophaga* (*Sarcophaga*) *lehmanni* (Muller, 1922)	2	0.06%	\	1	1	\	2	\
**\**	*Agria affinis* (Fallén, 1817)	2	0.06%	2	\	\	\	\	2
**\**	*Sarcophaga* (*Heteronychia*) *dissimilis* Meigen, 1826	1	0.03%	\	\	\	1	\	1
**\**	*Sarcophaga* (*Pandelleisca*) *similis* Meade, 1876	1	0.03%	\	\	1	\	1	0
**\**	*Macronychia polyodon* (Meigen, 1824)	1	0.03%	1	\	\	\	\	1
	**Total**	**3284**	**100%**	**1147**	**894**	**383**	**860**	**1276**	**2008**
		**N° species→**		**27**	**21**	**12**	**24**	**23**	**32**
