# Correction: Bonelli et al. Ecological Preferences of Calliphoridae and Sarcophagidae (Diptera) in the Region Calabria (Southern Italy). *Insects* 2025, *16*, 886

**DOI:** 10.3390/insects16101017

**Published:** 2025-10-01

**Authors:** Domenico Bonelli, Federica Mendicino, Francesco Carlomagno, Giuseppe Luzzi, Antonino Siclari, Federica Fumo, Erica Di Biase, Michele Mistri, Daniel Whitmore, Cristina Munari, Marco Pezzi, Teresa Bonacci

**Affiliations:** 1 Department of Biology, Ecology and Earth Sciences, University of Calabria, Via Pietro Bucci, 87036 Rende, Italy; domenico.bonelli@unical.it (D.B.); federica.mendicino@unical.it (F.M.); francesco.carlomagno@unical.it (F.C.); fumofede@gmail.com (F.F.); ericadibia@gmail.com (E.D.B.); teresa.bonacci@unical.it (T.B.); 2 Ente Parco Nazionale Lucano, Val d’Agri-Lagonegrese, Via Manzoni, 1, 85052 Marsico Nuovo, Italy; direttore@parcoappenninolucano.it; 3 Città Metropolitana di Reggio Calabria, Piazza Italia, 89100 Reggio Calabria, Italy; antonino.siclari@cittametropolitana.rc.it; 4 Department of Chemical, Pharmaceutical and Agricultural Sciences, University of Ferrara, Via Luigi Borsari 46, 44121 Ferrara, Italy; michele.mistri@unife.it (M.M.); cristina.munari@unife.it (C.M.); 5 Staatliches Museum für Naturkunde Stuttgart, Rosenstein 1, 70191 Stuttgart, Germany; daniel.whitmore@smns-bw.de; 6 Sistema Museale Universitario—SiMU, Sezione di Zoologia, University of Calabria, 87036 Rende, Italy

Daniel Whitmore was not included as an author in the original publication [1]. The corrected Author Contributions statement and Acknowledgements appears below. The authors state that the scientific conclusions are unaffected. This correction was approved by the Academic Editor. The original publication has also been updated.

Conceptualization, D.B., M.P. and T.B.; Data curation, D.B., F.M., F.C., G.L., A.S., F.F., E.D.B., M.M., C.M., M.P. and T.B.; Formal analysis, D.B., M.P. and T.B.; Investigation, D.B., F.M., F.C., F.F., E.D.B., D.W., M.P. and T.B.; Methodology, D.B., M.P. and T.B.; Supervision, T.B.; Validation, M.P. and T.B.; Writing—original draft, D.B., M.P. and T.B.; Writing—review and editing, D.B., G.L., A.S., M.M., C.M., M.P. and T.B. All authors have read and agreed to the published version of the manuscript.

The authors wish to thank the authorities of the Aspromonte National Park, of the Sila National Park, and of the Natural Regional Park of Serre (Calabria, Italy) for allowing us to conduct the monitoring project on Diptera Brachycera in these areas. Thanks are also due to Krzysztof Szpila (Nicolaus Copernicus University, Toruń, Poland), Domenico Gargano (University of Calabria), Salvatore Vavalà (Natural Regional Park of Serre), and Simone Rovito (University of Calabria). The authors wish to dedicate this study to the late renowned zoologist of the Department of Biology, Ecology and Earth Sciences, University of Calabria: Tullia Zetto Brandmayr (1949–2010).
